# Drug–Target Interaction Prediction via Dual-Interaction Fusion

**DOI:** 10.3390/molecules31030498

**Published:** 2026-01-31

**Authors:** Xingyang Li, Zepeng Li, Bo Wei, Yuni Zeng

**Affiliations:** School of Computer Science and Technology, Zhejiang Sci-Tech University, Hangzhou 310018, China; 2023337621278@mails.zstu.edu.cn (X.L.); zepengli56@gmail.com (Z.L.); weibo@zstu.edu.cn (B.W.)

**Keywords:** drug–target interaction prediction, multi-scale representation learning, dual-interaction fusion

## Abstract

Accurate prediction of drug–target interaction (DTI) is crucial for modern drug discovery. However, experimental assays are costly, and many existing computational models still face challenges in capturing multi-scale features, fusing cross-modal information, and modeling fine-grained drug–protein interactions. To address these challenges, We propose Gated-Attention Dual-Fusion Drug–Target Interaction (GADFDTI), whose core contribution is a fusion module that constructs an explicit atom–residue similarity field, refines it with a lightweight 2D neighborhood operator, and performs gated bidirectional aggregation to obtain interaction-aware representations. To provide strong and width-aligned unimodal inputs to this fusion module, we integrate a compact multi-scale dense GCN for drug graphs and a masked multi-scale self-attention protein encoder augmented by a narrow 1D-CNN branch for local motif aggregation. Experiments on two benchmarks, **Human** and ***C. elegans***, show that GADFDTI consistently outperforms several recently proposed DTI models, achieving AUC values of **0.986** and **0.996**, respectively, with corresponding gains in precision and recall. A SARS-CoV-2 case study further demonstrates that GADFDTI can reliably prioritize clinically supported antiviral agents while suppressing inactive compounds, indicating its potential as an efficient in silico prescreening tool for lead-target discovery.

## 1. Introduction

In the process of drug development, accurately evaluating drug–target interactions (DTIs) is crucial for drug efficacy and safety [1,2]. While traditional experimental methods for measuring drug–target interactions yield reliable results, they require substantial investment of human resources, materials, and finances [3]. Therefore, employing computational methods to predict drug–target interactions has become an essential research approach [4]. Recent advancements in sequence and graph representation learning methods have propelled progress in this field [1,5]. For instance, TransformerCPI demonstrates that its attention mechanism can be “deconvolved to highlight important interacting regions of protein sequences and compound atoms” [6]. MolTrans achieves superior generalization performance on unseen samples through its Transformer framework enhanced with substructure modeling focused on interaction semantics [7]. However, existing approaches still exhibit two key shortcomings. **(i) Insufficient encoding of drug graphs and protein sequences.** On the drug side, deep GNN encoders help capture long-range dependencies on molecular graphs but easily suffer from oversmoothing [8], where node representations lose discriminability and functional-group cues vanish as layers increase. Recent DTA/DTI models therefore either stay shallow or rely on dense skip connections and multi-scale aggregation; for example, MGraphDTA, which builds a 27-layer GNN with dense retention of intermediate states [9]. However, many pipelines still fuse scales in a coarse manner and do not explicitly balance shallow local chemistry and deep global topology. On the protein side, 1D-CNN- or vanilla Transformer-based encoders tend to emphasize either local motifs or global context, and can become unstable for long sequences. Multi-scale self-attention designs such as MSSAN partially alleviate this issue [10], but existing DTI models rarely integrate multi-scale attention with lightweight convolutional aggregation in a unified, width-aligned encoder for both drugs and targets. **(ii) Limited cross-modal fusion.** Many DTI studies still encode drugs and proteins independently and fuse them by simple concatenation or single-pass cross-attention, which weakens cross-modal coupling and hides interaction patterns in high-dimensional vectors. Even multimodal co-attention and knowledge-graph methods such as CoaDTI and BridgeDPI [11,12] mainly operate at the channel or entity level and do not explicitly construct atom–residue similarity fields. Consequently, they typically lack (a) a computable, fine-grained similarity map over atom–residue pairs and (b) fragment-level consistency constraints such as 2D neighborhood regularization and channel-wise gating. Under stricter splitting protocols and credible negative sampling [13], these limitations can lead to local false alignments, fluctuations in threshold-dependent metrics, and difficulties in explaining which atom–residue segments truly contribute to the predicted interaction [11,12,13,14].

To address the aforementioned gaps, we propose a Gated-Attention Dual-Fusion Drug–Target Interaction model (GADFDTI). First, we design a multi-scale dense connected GCN for drug graphs that retains cross-layer information and linearly compresses widths, mitigating over-smoothing while preserving functional-group clues and global topological insights. Second, we employ multi-scale masked self-attention [15] on proteins, combined with a lightweight 1D-CNN aggregator to balance motif-level locality and long-range dependencies while enhancing training stability. Third, we propose an attention-guided dual-interaction fusion block to predict DTIs. To the end, we evaluate the effectiveness of our proposed model on two benchmark datasets, **Human** and ***C. elegans***. Experimental results demonstrate that our GADFDTI model yields better accuracy over baselines and existing DTI deep models.

In this work, our main focus is the dual-interaction fusion block; the unimodal encoders are organized as a compact, width-aligned integration to provide strong atom/residue features for the proposed fusion mechanism under the canonical benchmark setting.

### Contributions

1. **Attention-guided dual-interaction fusion with explicit similarity field and 2D neighborhood refinement (primary contribution).** We construct an atom–residue similarity field, refine it with a lightweight 2D neighborhood operator, and perform gated bidirectional aggregation to couple drug and protein representations, encouraging fragment-level coherence and enabling visualization-friendly similarity maps.
2. **Width-aligned improvement of established multi-scale encoders (supporting contribution).** We propose a compact multi-scale dense GCN for drug graphs and a masked multi-scale self-attention protein encoder augmented with a narrow 1D-CNN branch, producing width-aligned atom/residue features that interface cleanly with the proposed fusion module.

## 2. Results

### 2.1. Datasets, Splits, and Metrics

This study utilized two publicly available drug–target interaction (DTI) datasets, namely, **Human** and ***C. elegans***. The positive samples in both datasets were derived from the authoritative **Matador** [16] and **DrugBank** [17] databases. Duplicate entries were removed prior to experiments to ensure evaluation integrity. Experiments were conducted on two public, class-balanced DTI benchmarks, **Human** and ***C. elegans***, whose sizes are summarized in Table 1. Following the canonical benchmark protocol, we adopt random splits at the interaction-pair level with an 8:1:1 train/validation/test ratio. We note that this setting evaluates performance in a warm-start scenario, where the same drug and/or target may appear in both training and test sets, which can yield optimistic estimates compared with real-world discovery settings. We therefore explicitly acknowledge this limitation and interpret our results under this protocol accordingly and discuss out-of-distribution generalization as future work. To reduce randomness and strengthen reliability, all experiments are repeated under 10 random seeds, and results are reported as mean ± standard deviation; in addition, two-sided paired *t*-tests across seeds are conducted when comparing GADFDTI to baselines. The decision threshold is determined on the validation set using the maximum F1 criterion and then fixed for test evaluation. Evaluation metrics include AUC, AUPR, Accuracy (ACC), Precision, Recall, and F1. Following recommendations on negative sampling, credible negatives are used to reduce potential label leakage and “easy negative” bias [13]. Key training and model hyperparameters are listed in Table 2.

Computational cost note: we report the resource setting for our implementation (batch size, epochs, and GPU memory). Training-time comparisons across different published methods are not directly comparable due to heterogeneous implementations, hardware, and optimization settings; therefore, we do not claim a fair cross-method timing benchmark in this work.

### 2.2. Experiments and Analysis

#### 2.2.1. Contribution of the Protein-Side 1D-CNN

We evaluated the impact of a stacked three-layer lightweight 1D-CNN on the multiscale multi-head self-attention encoder for capturing multiscale features in protein sequences.

The GADFDTI model in this study is constructed based on the MGNN method proposed by Yang [14] and the MSSAN method proposed by Zeng [10], so this experiment combined these two methods as baseline models. A three-layer 1D-CNN was then inserted into the protein branch, with all other settings unchanged.

Table 3 shows the results. On **Human**, adding 1D-CNN led to an increase in AUC, Precision and Recall rising by 0.001, 0.004 and 0.006, respectively. On ***C. elegans***, compared to baseline method, our model decreases by 0.001 for AUC, but has an increment, like 0.005 for Precision and 0.002 for Recall. This suggests that the CNN branch may trade a very small AUC decrease for improved Precision/Recall in some settings, and its benefit can be dataset dependent.

The gated interaction module generates adaptive weights based on the holistic semantic features of drugs and proteins. This effectively guides the dot-product interaction module to focus on critical drug–target binding regions, mitigating attention drift issues while efficiently integrating multi-scale features encompassing different levels of drugs and proteins. Consequently, employing the gated interaction module as a steering head to guide the dot-product interaction process represents an effective fusion strategy.

#### 2.2.2. Attention-Guided Dual Interaction (Gating + Dot-Product + Neighborhood)

We evaluate the contribution of each component in the proposed dual-interaction fusion module. We compare three variants: (i) gating only; (ii) dot-product only; and (iii) complete GADFDTI. We further remove the neighborhood refinement (-Neighborhood) to assess its effect. As shown in Table 4 and Table 5, the full configuration achieves the best overall performance on both benchmarks, while using only one interaction mechanism leads to slightly lower AUC/Precision/Recall. Removing the neighborhood refinement results in modest but consistent drops, indicating that local smoothing on the similarity field is generally beneficial.

The gated interaction and dot-product interaction are complementary; gating provides a coarse global guide, while the explicit similarity field captures fine-grained atom–residue matches. Combining them tends to yield more balanced Precision/Recall than either component alone under the canonical split.

The neighborhood refinement tends to help more on the smaller dataset, suggesting that a lightweight coherence prior can reduce noise in sparse interaction patterns; nevertheless, we do not claim universal gains for every dataset or split.

#### 2.2.3. Comparison of Fusion Mechanisms

Compare GADFDTI with various other attention fusion designs in terms of accuracy and the other test evaluation metrics. GADFDTI is replaced with Cross Attention [18], Bi-directional Intentional Attention [19], Joint Attention [20], and Bilinear Attention [21], while keeping the backbone and training protocol fixed. Metrics are reported on both datasets. GADFDTI yields the best AUC/Recall combination on both benchmarks (Table 6). On **Human**, it reaches AUC 0.986 and Recall 0.960 while maintaining the highest Precision. On ***C. elegans***, it attains AUC 0.996 and Recall 0.967. Bi-intent attention exhibits slightly higher Precision on ***C. elegans*** but with a clear loss in Recall (Table 6).

Results demonstrate that on **Human**, the dual-interaction feature fusion method proposed in this study achieves the best performance among all fusion methods. On ***C. elegans***, this method achieves optimal performance on both metrics except for Precision, where it slightly underperforms the bidirectional intent attention method. This further highlights the superiority of the fusion method proposed in our study.

#### 2.2.4. Classification Comparison Experiment

Compare the GADFDTI method against six representative drug–target interaction prediction models from the past five years across two public datasets—**Human** and ***C. elegans***—we assess the relative performance of GADFDTI under the same experimental protocol.

All methods take drug graph structures and protein sequences as inputs, with experiments configured as binary classification tasks. Evaluation metrics include AUC, Precision, and Recall. Selected comparison models comprise TransformerCPI [6], MHSADTI [22], CoaDTI [11], Mutual-DTI [23], MdDTI [24], and MultiGranDTI [9], representing mainstream modeling approaches in recent years across feature expression, attention mechanisms, and graph neural networks.

On **Human**, GADFDTI achieved the best performance on both Precision and Recall metrics, reaching 0.966 and 0.960, respectively. Compared to the other six methods, Precision improved by 0.4% to 4.8%, while Recall increased by 0.4% to 3%. Although GADFDTI slightly underperformed MdDTI in AUC, it achieved substantial gains in Precision and Recall. On ***C. elegans***, GADFDTI outperformed other methods across all evaluation metrics except AUC. It improved AUC and Precision by 0.2–1.3% and 1.1–3.4%, respectively, while Recall was marginally lower than MdDTI (by 0.3%), but surpassed the other two methods by 0.2% and 1.1% in Precision and Recall, respectively, lower than the MdDTI method (by 0.3%), but the other two metrics were higher by 0.2% and 1.1%, respectively (Table 7).

Table 7 summarizes the classification performance. On Human, GADFDTI achieves high Precision and Recall (0.966/0.960), while its AUC is close to the best-performing baseline. On *C. elegans*, GADFDTI attains 0.996 AUC with 0.975 Precision and 0.967 Recall, remaining competitive with the strongest competitors. Overall improvements over other baselines are typically modest (e.g., 0.1–0.3% AUC and ~0.4–1% Precision/Recall) but overall performance was still improved and consistent across different random seeds.

To quantify robustness, we report results over 10 random seeds and perform two-sided paired *t*-tests across matched seeds (Section 2.2.7; Supplementary Table S1). Under the canonical split, GADFDTI significantly outperforms most baselines, whereas differences to the strongest competitors are not always statistically significant.

**Experimental and statistical settings.** Results are reported as mean ± standard deviation over 10 random seeds using the same 8:1:1 train/validation/test split and identical hyperparameter settings. The classification threshold is selected on the validation set by the maximum F1 criterion and then fixed for test evaluation. Statistical significance between GADFDTI and each baseline is assessed using a two-sided paired *t*-test across matched seeds (*n* = 10 means set 10 random seeds, α = 0.05 means significance level is 0.05). Full *p*-values are provided in Supplementary Table S1.

#### 2.2.5. Illustrative Case Study: SARS-CoV-2 Targets

We provide a small illustrative case study to demonstrate how the model can be used to rank candidate compounds for a target, rather than a structural validation of binding sites.

Two viral targets, 3CL_pro_ and RdRp, are considered. Following the candidate list in prior work [12], we selected eight representative drugs (e.g., Baricitinib and Ivermectin) and used GADFDTI to predict interaction probabilities for these target–drug pairs. The goal is to illustrate the model’s ranking behavior in a practical prescreening setting.

First, the amino acid sequences for 3CL_pro_ and RdRp were obtained from the Protein Data Bank (PDB) and the National Center for Biotechnology Information (NCBI), respectively. The SMILES sequences for the eight candidate drugs were extracted from the Drug Bank database. Subsequently, these amino acid sequences and SMILES sequences were input into the GADFDTI model for prediction, ultimately yielding their interaction probabilities.

Baricitinib, Remdesivir, Ritonavir, and Lopinavir all exhibit predicted likelihoods above 0.8 for 3CL_pro_, exceeding the model’s probability threshold of 0.5. Meanwhile, Ivermectin, Sofosbuvir, Remdesivir, Daclatasvir, Lopinavir, and Ritonavir all show predicted likelihoods above 0.5 for RdRp. Aspirin exhibits a low probability of interaction with both 3CL_pro_ and RdRp (Table 8).

Baricitinib, Remdesivir, Ritonavir, and Lopinavir are all drugs with high potential for interaction with 3CL_pro_ [25,26]. Meanwhile, Ivermectin, Sofosbuvir, Remdesivir, Daclatasvir, Lopinavir, and Ritonavir are predicted to bind effectively with RdRp [25,26]. The results predicted by the GADFDTI model align with findings validated by numerous studies and clinical trials.

Conversely, Aspirin fails to inhibit either the 3CL_pro_ or RdRp targets, rendering it ineffective for treating target-related diseases—a finding consistent with real-world observations.

Overall, this small case study suggests that GADFDTI can produce qualitatively plausible rankings consistent with prior reports.

#### 2.2.6. Generalizability and Scalability Limitations

Our evaluation follows the canonical interaction-pair random split and thus primarily reflects warm-start performance. Generalization to unseen targets, novel chemical scaffolds, or fully cold-start scenarios (e.g., cold-drug/cold-target, scaffold split, or protein-family held-out) is not measured in this study and may be more challenging; we leave such out-of-distribution evaluation for future work. Although the explicit atom–residue similarity field enables post hoc visualization, we do not provide quantitative PDB-aligned binding-site validation or overlap metrics, which also remains future work. Regarding scalability, the dual-interaction similarity field grows with the numbers of atoms and residues; while the neighborhood refinement is lightweight, memory/time may increase for long proteins or large molecules. We therefore report computational statistics (training epochs, GPU memory usage, and batch size). For long proteins, we additionally report an inference-time sliding-window aggregation result on the >1200 subset (window = 1200, stride = 300, max pooling; Supplementary Table S2), which yields modest but consistent gains over simple truncation. We note that residue-level preservation of binding regions cannot be rigorously verified under these canonical benchmarks due to the lack of structure-aligned binding-site annotations; structure/PDB-aligned validation remains future work.

Although this study focuses on small-molecule–protein DTI, the proposed dual-interaction fusion is, in principle, modality-agnostic as long as token-/node-level representations are available on both sides. For protein–DNA or protein–RNA systems, the protein branch can be kept while the nucleic-acid side can be encoded by a sequence encoder (k-mer embedding + masked self-attention/CNN) or a structure-aware encoder if secondary/tertiary information is available. For DNA/RNA–small molecule interactions, the drug graph encoder can be reused, and the nucleic-acid sequence encoder can replace the protein encoder, while the atom–token similarity field and neighborhood refinement remain applicable.

#### 2.2.7. Statistical Testing Across Seeds

Over 10 random seeds, GADFDTI achieves 0.9866 ± 0.0020 AUC on Human and 0.9962 ± 0.0020 on *C. elegans*. Using two-sided paired *t*-tests across seeds (α = 0.05), GADFDTI shows statistically significant improvements over many baseline methods under the canonical split, while differences versus the strongest competitors are not always significant. In particular, on Human the differences versus Mutual-DTI (*p* = 0.0683) and MdDTI (*p* = 0.1693) are not significant; on *C. elegans*, comparisons versus Baseline + 1D-CNN (*p* = 0.2300) and the “-Neighborhood” variant (*p* = 0.1612) are also not significant. Full paired *t*-test results (t statistic, *p*-value, and mean difference across 10 matched seeds) are provided in Supplementary Table S1.

## 3. Materials and Methods

### 3.1. Task Definition

**Figure 1:**
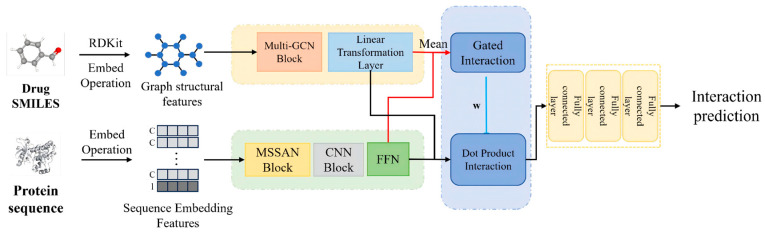
Overview of GADFDTI. The drug and protein encoders produce width-aligned features ${X_{D}}^{\prime },{X_{P}}^{\prime }$ and global summaries $z_{D},z_{P}$. The dual-interaction fusion module constructs an atom–residue similarity field, applies 2D neighborhood convolution, performs bidirectional aggregation, and uses channel-wise gating to obtain fused vectors, which are finally mapped to the interaction probability p.

In this work, we formulate drug–target interaction prediction as a binary classification problem. Given a drug molecule and a target protein, the goal is to predict whether they interact by outputting an interaction probability. The proposed GADFDTI model consists of four modules: a multi-scale graph convolutional network (GCN) drug encoder, a multi-scale multi-head self-attention protein encoder, a dual-interaction fusion module, and an interaction prediction module. The overall architecture is illustrated in Figure 1. In summary, the drug’s SMILES string is first converted into a molecular graph and encoded by the multi-scale GCN to capture local and global structural features. The protein’s amino acid sequence is encoded by a Transformer-based multi-scale attention encoder to capture local motifs and global context. The resulting multi-scale representations are then fused by a dual-interaction mechanism that combines a gating-based interaction and a dot-product attention-based interaction, effectively modeling the complex relationships between the drug and target. Finally, the fused representation is passed through a prediction network to output the probability of interaction.

Each sample consists of a drug molecular graph G=(V,E) with |V|=n atoms and bonds E, and a protein sequence $S=\{a_{1},\ldots ,a_{\ell }\}$ of length ℓ. Given (G,S), the model with parameters θ outputs a logit $s=f_{\theta }(G,S)\in R$ and a probability p=σ(s)∈[0,1], where $\sigma (u)=1/(1+e^{-u})$. Predictions are trained under a binary cross-entropy loss over positive and negative drug–target pairs.

### 3.2. Model Overview

GADFDTI comprises three stages (Figure 1): (i) single-modality encoding, where a graph encoder produces atom-level drug features and a sequence encoder produces residue-level protein features; (ii) attention-guided dual-interaction fusion, where an explicit atom–residue similarity field is constructed, locally enhanced, and used for bidirectional gated fusion; and (iii) prediction, where pooled representations are mapped to the DTI probability.

From raw inputs we build $X_{D}^{\left(0\right)}$ (atom features from G) and $X_{P}^{\left(0\right)}$ (residue embeddings from S). The drug encoder maps $\left(X_{D}^{\left(0\right)},G\right)$ to an atom-level representation ${X_{D}}^{\prime }\in R^{n\times m}$ and a graph summary $z_{D}\in R^{m}$; the protein encoder maps $X_{P}^{\left(0\right)}$ to a residue-level representation ${X_{P}}^{\prime }\in R^{\ell \times m}$ and a sequence summary $z_{P}\in R^{m}$. These four tensors $\left\{{X_{D}}^{\prime },{X_{P}}^{\prime },z_{D},z_{P}\right\}$ form the interface to the fusion module, whose outputs are then fed into a two-layer MLP decoder.

### 3.3. Input Construction and Feature Representation

**Input Representation.** The model takes as input a drug SMILES string and a target protein FASTA sequence, which are processed into suitable formats for the encoders. For each drug, we use the RDKit toolkit to convert the SMILES string into a molecular graph G=(V,E), where V is the set of atoms and E is the set of bonds [27]. Each atom $v_{i}\in V$ is represented as a node and each bond $e_{ij}\in E$ as an edge between atoms i and j. We initialize every atom with an feature vector capturing its chemical properties. In particular, we encode eight categories of atomic attributes (atom type, degree, number of attached hydrogens, implicit valence, hybridization, aromaticity, chirality, and chirality type) using one-hot or binary encodings, resulting in an 87-dimensional feature vector for each atom [28]. For each protein, we take its amino acid sequence (in FASTA format) as input. Each amino acid is mapped to a learnable 128-dimensional embedding vector, and a positional encoding is added to incorporate sequence order [29]. We set a maximum sequence length of 1200 (truncating any longer sequences) to cover about 80% of proteins in our data for efficient training [30]. We note that truncation may affect a subset of long-protein pairs. To mitigate this issue without changing the training protocol, we additionally evaluate an inference-time sliding-window aggregation strategy for targets longer than 1200 residues. Specifically, a long sequence is segmented into overlapping windows of length 1200 with stride 300, and the final DTI score is obtained by max pooling over window-level predictions. This evaluation is performed at inference only (no retraining) to keep the training setting consistent; results on the >1200 subset are reported in Supplementary Table S2. This strategy yields modest but consistent improvements over simple truncation on the >1200 subset for both benchmarks.

Given a drug–target pair (G,S), as defined in Section 3.1, we construct the initial feature matrices, we construct the initial feature matrices $X_{D}^{\left(0\right)}\in R^{n\times d_{D}}$ and $X_{P}^{\left(0\right)}\in R^{\ell \times d_{P}}$, together with auxiliary structures for the encoders.

#### 3.3.1. Drug Side: From SMILES to Atom Features

Each drug is provided as a SMILES string and parsed by RDKit into an undirected molecular graph G=(V,E) with |V|=n atoms and bonds E. For each atom $v_{i}\in V$ we construct a feature vector $x_{i}^{\left(D\right)}\in R^{d_{D}}$ using standard RDKit descriptors, including element type, degree, valence, formal charge, hybridization, aromaticity, ring membership, attached hydrogens, simple structural flags (e.g., conjugation, multi-ring membership), and a few normalized scalar attributes such as scaled atomic mass. Stacking all atom features yields $X_{D}^{\left(0\right)}=[x_{1}^{\left(D\right)};\ldots ;x_{n}^{\left(D\right)}]\in R^{n\times d_{D}}$. We also construct an adjacency matrix $A\in \{0,1{\}}^{n\times n}$ (or edge index list) from the bond set, optionally augmented with bond-type encodings for edge-aware normalization. The drug encoder (Section 3.4) takes $\left(X_{D}^{\left(0\right)},A\right)$ and outputs ${X_{D}}^{\prime }$ and $z_{D}$.

#### 3.3.2. Protein Side: From Amino-Acid Sequence to Residue Embeddings

Each protein is given as an amino-acid sequence $S=\{a_{1},\ldots ,a_{\ell }\}$. Characters are mapped to indices over a vocabulary containing 20 standard amino acids plus tokens for unknown residues and padding. Sequences longer than a preset maximum length $L_{max}$ (Table 2) are truncated; shorter sequences are right padded to $L_{max}$ and accompanied by a padding mask.

A trainable embedding matrix $E_{P}\in R^{|V_{P}|\times d_{P}}$ maps token indices to vectors, forming $X_{P}^{\left(0\right)}\in R^{\ell \times d_{P}}$ (with $\ell \leq L_{max}$). Positional encodings (sinusoidal or learned) are optionally added. The protein encoder (Section 3.5) takes $X_{P}^{\left(0\right)}$ and the mask, and outputs ${X_{P}}^{\prime }$ and $z_{P}$.

#### 3.3.3. Interface to Encoders

In summary, raw inputs are converted once into

$$X_{D}^{\left(0\right)},\;A\;and\;X_{P}^{\left(0\right)},\;mask,$$

with fixed dimensions $\left(d_{D},d_{P}\right)$. The encoders map these to width-aligned latent representations ${X_{D}}^{\prime }\in R^{n\times m}$ and ${X_{P}}^{\prime }\in R^{\ell \times m}$ and to global summaries $z_{D},z_{P}\in R^{m}$, which exactly match the interface used by the fusion module in Section 3.6.

### 3.4. Drug Encoder: Multi-Scale Densely Connected GCN

**Figure 2:**
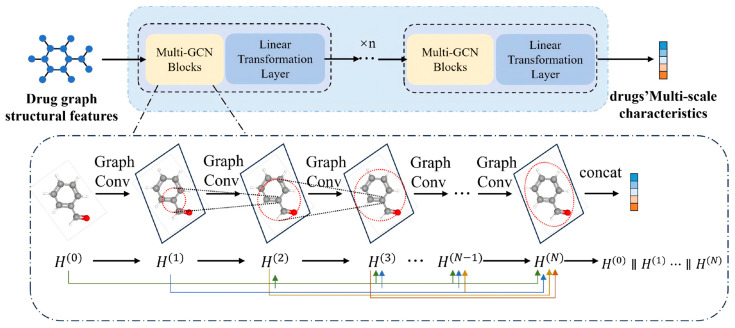
Drug encoder. Each block performs multi-scale dense retention and GCN aggregation (Equations (1)–(3)), then projects to a shared width m via a linear transition layer (Equation (4)). Stacking B blocks produces atom-level features ${X_{D}}^{\prime }$ and a graph summary $z_{D}$ for the fusion module.

The drug encoder is designed to capture both local functional-group signals and global graph topology while alleviating oversmoothing in deep graph convolutional networks (GCNs). Simply stacking many GCN layers enlarges the receptive field but often leads to vanishing gradients and oversmoothed node features, where atom representations become indistinguishable and functional-group cues are washed out. To avoid this, we adopt a multi-scale, densely connected GCN architecture inspired by the MGNN-style design in recent multiscale graph models [31,32]. As illustrated in Figure 2, the encoder consists of B stacked blocks; each block applies L, graph convolutional layers with multi-scale dense retention, followed by a linear “width alignment” layer that compresses concatenated features back to a fixed channel width m, Given initial atom features $X_{D}^{\left(0\right)}\in R^{n\times d_{D}}$ for a molecular graph G=(V,E) with n=|V| atoms and adjacency matrix A, the encoder outputs node-level representations ${X_{D}}^{\prime }\in R^{n\times m}$ and a graph-level summary $z_{D}\in R^{m}$, which are fed into the fusion module (Figure 2).

#### 3.4.1. Within-Block Computation

Consider a single block (block index $b$ omitted unless needed). Let

$$H^{\left(\ell \right)}=[h_{1}^{\left(\ell \right)};\ldots ;h_{n}^{\left(\ell \right)}]\in R^{n\times M}$$

denote the node feature matrix after the ℓ-th GCN layer in this block (ℓ=0,…,L), where $h_{i}^{\left(\ell \right)}\in R^{M}$ is the feature vector of atom i and $H^{\left(0\right)}$ is the block input ($H^{\left(0\right)}=X_{D}^{\left(0\right)}$ for the first block and $H^{\left(0\right)}=Z_{b-1}$ for subsequent blocks). To implement multi-scale dense retention, each layer first concatenates all intermediate states from 0 to ℓ:

(1) $${\hat{h}}_{i}^{\left(\ell \right)}=h_{i}^{\left(0\right)}\;\|\;h_{i}^{\left(1\right)}\;\|\;\cdots \;\|\;h_{i}^{\left(\ell \right)},{\hat{H}}^{\left(\ell \right)}=Concat(H^{\left(0\right)},H^{\left(1\right)},\ldots ,H^{\left(\ell \right)}),$$

where ‖ denotes channel-wise concatenation along the feature dimension and ${\hat{H}}^{\left(\ell \right)}\in R^{n\times d_{cat}^{\left(\ell \right)}}$; for brevity we write $d_{cat}$ when the dimension is clear from context.

At a high level, the (ℓ+1)-th GCN layer within the block can be summarized compactly as

(2) $$H^{\left(\ell +1\right)}=GCN([H^{\left(0\right)}\;\|\;H^{\left(1\right)}\;\|\;\cdots \;\|\;H^{\left(\ell \right)}],\;\Theta_{\ell +1}),$$

where $\Theta_{\ell +1}$ collects all trainable parameters of the (ℓ+1)-th layer and GCN(⋅) denotes a generic graph convolution operator acting on the concatenated multi-scale features. This view emphasizes that each layer has access to both shallow and deep representations [33,34].

Concretely, we instantiate GCN(⋅)  as a first-order graph convolution on the concatenated features. The (ℓ+1)-th layer updates each node i as

(3) $$h_{i}^{\left(\ell +1\right)}=\phi (\Phi_{1}\;{\hat{h}}_{i}^{\left(\ell \right)}+\Phi_{2}\;{\sum_{j\in N(i)}{\hat{h}}_{j}}^{\left(\ell \right)}),$$

where N(i)⊆V is the neighbor set of node i in G, $\Phi_{1},\Phi_{2}\in R^{d_{cat}\times M}$ are learnable weight matrices for self- and neighbor-messages, respectively, and ϕ(⋅) is a pointwise nonlinearity (e.g., ReLU). Because each layer operates on the concatenated ${\hat{H}}^{\left(\ell \right)}$, shallow and deep features remain accessible throughout the block, which helps mitigate oversmoothing in deep GCNs.

#### 3.4.2. Block Output and Multi-Block Stacking

After L layers in block b, we again concatenate all intermediate states and project them to the shared width m via a linear transition (width alignment) layer:

(4) $$Z_{b}=\phi (Concat(H_{b}^{\left(0\right)},H_{b}^{\left(1\right)},\ldots ,H_{b}^{\left(L\right)})\;W_{b}^{lin})\in R^{n\times m},$$

where $H_{b}^{\left(\ell \right)}$ denotes the feature matrix of block b at layer ℓ, $W_{b}^{lin}\in R^{d_{cat}\times m}$ is a block-specific projection matrix, and the nonlinearity ϕ(⋅) is reused. This linear transition compresses the concatenated multi-scale features to a fixed width m, controlling the model size while retaining information from all layers in the block.

The full drug encoder stacks B such blocks in series. The first block takes $X_{D}^{\left(0\right)}$ as input and outputs $Z_{1}$; block b takes $Z_{b-1}$ as input and outputs $Z_{b}$. The final node-level representation is

$${X_{D}}^{\prime }=Z_{B}\in R^{n\times m},$$

where each row of ${X_{D}}^{\prime }$ is the m-dimensional embedding of one atom.

A permutation-invariant readout (mean pooling in our implementation) then produces a compact graph-level summary:

$$z_{D}=Readout({X_{D}}^{\prime })=\frac{1}{n}\sum_{i=1}^{n}{x_{i}}^{\prime (D)}\in R^{m},$$

where ${x_{i}}^{\prime (D)}$ is the i-th row of ${X_{D}}^{\prime }$ and Readout(⋅) denotes averaging over the node dimension.

#### 3.4.3. Summary of Drug Encoder

In summary, the drug encoder implements

$$(X_{D}^{\left(0\right)},A)⟼{X_{D}}^{\prime }\in R^{n\times m},z_{D}\in R^{m}.$$

The combination of multi-scale dense retention and linear width alignment allows the model to keep shallow and deep atom features simultaneously under a fixed width m, providing both atom-level representations and a global molecular summary that are directly consumed by the dual-interaction fusion module.

### 3.5. Protein Encoder: Multi-Scale Masked Self-Attention with Lightweight 1D-CNN

**Figure 3:**
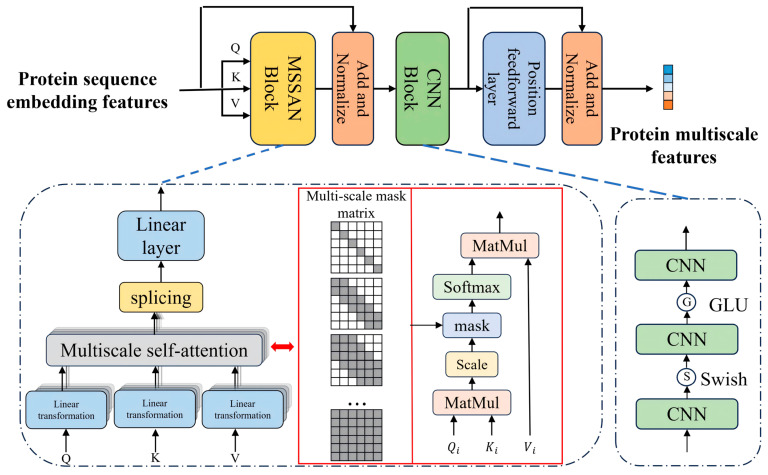
Protein encoder. A width-aligned projection maps initial residue features to dimension m, followed by multi-scale masked self-attention with four heads specializing in self, local, mid-range and global contexts. A lightweight 1D-CNN branch aggregates local patterns on top of the attention output. Their outputs are fused via normalized residual connections to produce residue-level features ${X_{P}}^{\prime }$ and a sequence summary $z_{P}$ for the fusion module.

The protein encoder follows the same design principle as the drug encoder; it aims to capture multi-scale sequence patterns with a compact, width-aligned architecture. Concretely, it combines an input projection, a multi-scale masked self-attention (MSSAN) [10] module, and a lightweight 1D-CNN branch. Compared with a vanilla Transformer encoder, our design introduces band-masked multi-head attention to explicitly separate local, mid-range and global contexts, and augments it with a very narrow CNN branch to aggregate high-order local motifs, which is particularly suitable for modeling binding sites of 3–10 amino acids within long protein sequences (up to length 1200) (Figure 3).

#### 3.5.1. Input Projection

To align channel width with the drug side, we first project the initial residue features to the shared width m:

(5) $$X_{P}=X_{P}^{\left(0\right)}W_{P}^{in}\in R^{\ell \times m},$$

where $X_{P}^{\left(0\right)}\in R^{\ell \times d_{P}}$ denotes the initial residue features (e.g., token embedding plus positional encoding) for a padded protein sequence of length ℓ, $d_{P}$ is the original feature dimension, and $W_{P}^{in}\in R^{d_{P}\times m}$ is a learnable projection matrix. A binary padding mask (“mask”) indicating valid residues is reused as part of the attention mask to prevent attending to padded positions.

#### 3.5.2. Multi-Scale Masked Self-Attention

We adopt multi-head self-attention with band masks at different window sizes (identity, local, mid-range, and global) following the MSSAN design [10]. Let H be the number of heads (in our implementation H=4) and let $m=H\cdot d_{h}$ where $d_{h}$ is the per-head dimension. For head h∈{1,…,H}, we compute query, key and value matrices as

(6) $$Q_{h}=X_{P}W_{h}^{Q},{\;\;\;\;K}_{h}=X_{P}W_{h}^{K},\;V_{h}=X_{P}W_{h}^{V},$$

where $W_{h}^{Q},W_{h}^{K},W_{h}^{V}\in R^{m\times d_{h}}$ are learnable projection matrices and $Q_{h},K_{h},V_{h}\in R^{\ell \times d_{h}}$.

To enforce different receptive fields, we construct a band mask $M^{\left(h\right)}\in R^{\ell \times \ell }$ for each head. For a residue at position i, only positions j with $|i-j|\leq m_{h}$ are allowed to attend, where the head-specific window $m_{h}$ is chosen as

$$m_{1}=0,\;m_{2}=3,\;m_{3}=12,\;m_{4}=\ell ,$$

corresponding to self-only, local (short-range), mid-range and global attention, respectively. Entries outside the allowed band or on padded positions are assigned a large negative value (e.g., −∞) so that they are effectively ignored after the softmax. Scaled dot-product attention for head h is then given by

(7) $$A_{h}=softmax\left(\frac{Q_{h}K_{h}^{⊤}}{\sqrt{d_{h}}}+M^{\left(h\right)}\right),O_{h}=A_{h}V_{h},$$

where $A_{h}\in R^{\ell \times \ell }$ are attention weights and $O_{h}\in R^{\ell \times d_{h}}$ is the head output. The band masks $M^{\left(h\right)}$ explicitly bias different heads towards patterns at different scales: head 1 captures purely positional/self information ($m_{1}=0$), head 2 focuses on local motifs (e.g., short binding segments with length 3–10), head 3 covers mid-range interactions, and head 4 attends globally to the whole sequence.

The outputs of all heads are concatenated and projected back to width m:

(8) $$\;O=[\;O_{1}\;\|\;O_{2}\;\|\;\cdots \;\|\;O_{H}\;]W^{O},$$

where [⋅‖⋅] denotes concatenation along the feature dimension, $[\;O_{1}\|\cdots \|O_{H}\;]\in R^{\ell \times m}$ and $W^{O}\in R^{m\times m}$ is a learnable output projection. A residual connection with layer normalization then yields the multi-scale self-attention output:

(9) $$H_{msa}=LayerNorm(X_{P}+O)\in R^{\ell \times m}.$$

Here, LayerNorm(⋅) denotes channel-wise layer normalization. This design allows different heads to specialize in different sequence scales [35,36], while the residual connection stabilizes training and preserves the original embedding information.

#### 3.5.3. Lightweight 1D-CNN Aggregation

Although self-attention can capture long-range dependencies, convolutional filters are particularly effective at aggregating high-order local patterns and smoothing noise. To stabilize training and enhance local pattern aggregation, we append a narrow 1D-CNN branch on top of $H_{msa}$. Denoting $X_{N}=H_{msa}$ as the input to the CNN block, we apply a three-layer stack of depthwise 1D convolutions with small kernels (e.g., 3−5−3) and nonlinear activations:

(10) $$X_{C}={Conv}_{3}(\phi_{2}({Conv}_{2}(\phi_{1}({Conv}_{1}(X_{N}))))),$$

where ${Conv}_{i}$ denotes the i-th 1D convolution layer applied along the sequence dimension (with output width m), $\phi_{1}$ is the Swish activation and $\phi_{2}$ is a gated linear unit (GLU) activation applied to the intermediate layers [37,38]. Swish introduces smooth nonlinearity, while GLU acts as a soft gate to selectively pass informative channels and suppress noisy ones. The CNN branch produces $X_{C}\in R^{\ell \times m}$ (also denoted $H_{cnn}$), providing a locally smoothed and aggregated view of the sequence at negligible parameter overhead compared to the attention module.

#### 3.5.4. Encoder Output

The final residue-level representation is obtained by fusing the projected input $X_{P}$, the multi-scale self-attention output $H_{msa}$ and the CNN output $X_{C}$ via normalized residual connections:

(11) $${X_{P}}^{\prime }=LayerNorm(H_{msa}+X_{C})\in R^{\ell \times m},$$

where ${X_{P}}^{\prime }$ provides scale-aware residue embeddings that combine global context, multi-scale attention and local CNN aggregation. A permutation-invariant mean pooling over the sequence dimension then yields the protein summary:

$$z_{P}=MeanPool({X_{P}}^{\prime })=\frac{1}{\ell_{valid}}\sum_{i=1}^{\ell_{valid}}{x_{i}}^{\prime (P)}\in R^{m},$$

where ${x_{i}}^{\prime (P)}$ is the i-th row of ${X_{P}}^{\prime }$ corresponding to a valid (residue, non-padding) position and $\ell_{valid}$ is the number of valid residues in the sequence.

#### 3.5.5. Summary of Protein Encoder

Overall, the protein encoder implements

$$(X_{P}^{\left(0\right)},mask)⟼{X_{P}}^{\prime }\in R^{\ell \times m},\;z_{P}\in R^{m},$$

providing scale-aware residue representations and a compact global summary for the subsequent dual-interaction fusion module. Its main innovations lie in (i) explicitly modeling self, local, mid-range and global contexts via head-specific band masks, so that binding-site patterns at different scales are captured in a disentangled manner; (ii) using a lightweight 1D-CNN branch with Swish and GLU activations to aggregate higher-order local motifs without significantly increasing parameter count; and (iii) width alignment with the drug encoder, which simplifies cross-modal fusion.

### 3.6. Attention-Guided Dual-Interaction Fusion and Prediction

Given drug and protein encodings from the preceding encoders,

$${X_{P}}^{\prime }\in R^{n\times m},z_{D}\in R^{m},\;\;\;\;\;\;\;\;{X_{P}}^{\prime }\in R^{\ell \times m},z_{P}\in R^{m},$$

where n is the number of atoms in the drug molecular graph, ℓ is the length of the protein sequence, and m is the shared channel width, the fusion module couples the two modalities through a dual-interaction mechanism. Instead of directly concatenating $z_{D}$ and $z_{P}$ or naively attending over heterogeneous features, we explicitly (i) construct an atom–residue similarity field, (ii) enhance it by 2D neighborhood aggregation and bidirectional cross-attention, and (iii) perform channel-wise gated residual fusion guided by global summaries. A standard MLP decoder then maps the fused representation to the final interaction probability.

#### 3.6.1. Dot-Product Interaction: Explicit Similarity Field

We first construct an explicit atom–residue similarity matrix that serves as a “similarity field” for interaction modeling and visualization. Let $x_{i}^{\left(D\right)}\in R^{m}$ be the i-th row of ${X_{D}}^{\prime }$ (atom i) and $x_{j}^{\left(P\right)}\in R^{m}$ be the j-th row of ${X_{P}}^{\prime }$ (residue j). The scaled dot-product similarity is defined as

(12) $$\;\;\;I=\frac{1}{\sqrt{m}}\;{X_{D}}^{\prime }({X_{P}}^{\prime }{)}^{⊤}\in R^{n\times \ell },I_{ij}=\frac{\left\langle x_{i}^{\left(D\right)},\;x_{j}^{\left(P\right)}\right\rangle }{\sqrt{m}},$$

where ⟨⋅,⋅⟩ denotes the Euclidean inner product. A larger $I_{ij}$ indicates a stronger potential interaction between atom i and residue j. This explicit similarity field enables post hoc visualization of which atom–residue pairs contribute most strongly to the prediction; we use it as a qualitative interpretability aid.

#### 3.6.2. Neighborhood Interaction: 2D Enhancement and Bidirectional Aggregation

Drug–protein interactions are typically mediated by spatially contiguous substructures rather than isolated atoms or residues. To encourage fragment-level coherence on the similarity field, we apply a shallow 2D convolution over I:

(13) $$\;\;O=\psi ({Conv}_{2D}(I))\in R^{n\times \ell },$$

where ${Conv}_{2D}$ is a learnable 2D convolution with a small kernel (e.g., 3×3) and stride 1 over the atom and residue axes, and ψ(⋅) is a pointwise nonlinearity (e.g., ReLU). The resulting matrix O aggregates local neighborhoods on the atom–residue grid so that contiguous fragments form smoother activation patterns and noise is reduced.

Importantly, we do not assume that the atom and residue neighborhoods are geometrically aligned in a true 2D physical space. The atom–residue matrix is an interaction “field” indexed by atom order (from the molecular graph) and residue order (from the primary sequence), and the 2D neighborhood operator is introduced as a lightweight coherence/denoising inductive bias on similarity activations rather than a claim of chemical or biological grid alignment. More structure-aware alternatives (e.g., graph-aware smoothing, dilated kernels, or learned attention-based smoothing) are left for future work.

We then perform bidirectional aggregation to obtain context-enriched features on both sides. First, we normalize each row of O with a softmax to obtain a drug-to-protein attention distribution $A_{D}\in R^{n\times \ell }$:

(14) $$A_{D}(i,:)=Softmax(O(i,:)),$$

where $A_{D}(i,j)$ measures how strongly atom i attends to residue j. Similarly, we normalize each column of O to obtain a protein-to-drug attention distribution $A_{P}\in R^{n\times \ell }$:

(15) $$A_{P}(:,j)=Softmax(O(:,j)),$$

where $A_{P}(i,j)$ measures how strongly residue j attends to atom i. Using these directional attentions, we aggregate cross-modal context by weighted summation:

(16) $$X_{Dm}=A_{D}\;{X_{P}}^{\prime }\in R^{n\times m},$$

(17) $$X_{Pm}=A_{P}^{⊤}{X_{D}}^{\prime }\in R^{\ell \times m},$$

where $X_{Dm}$ represents drug-side features enriched with protein context, and $X_{Pm}$ represents protein-side features enriched with drug context. The subscript “m” highlights that these features are interaction informed.

#### 3.6.3. Gated Interaction: Channel-Wise Bidirectional Residual Fusion

Bidirectional aggregation injects cross-modal information at every atom and residue. However, drug and protein features may have different noise levels and importance across channels. To control the strength of cross-modal injection per channel, we derive a gating vector from the global summaries $z_{D}$ and $z_{P}$. We first concatenate them into a 2m-dimensional vector $[\;z_{D};z_{P}\;]\in R^{2m}$ and pass it through a small two-layer feed-forward network with batch normalization and ReLU, followed by a Sigmoid:

(18) $$\;\;\;w=\sigma ({BN}_{2}(L_{2}(\delta ({BN}_{1}(L_{1}([z_{D};z_{P}]))))))\in [0,1{]}^{m},$$

where $L_{1}:R^{2m}\to R^{m}$ and $L_{2}:R^{m}\to R^{m}$ are linear layers, ${BN}_{1},{BN}_{2}$ are batch normalization layers, δ(⋅) is a ReLU activation, and σ(⋅) is an element-wise Sigmoid. The k-th entry $w_{k}$ encodes how strongly the k-th channel should rely on cross-modal interaction information when updating the fused representation.

**Figure 4:**
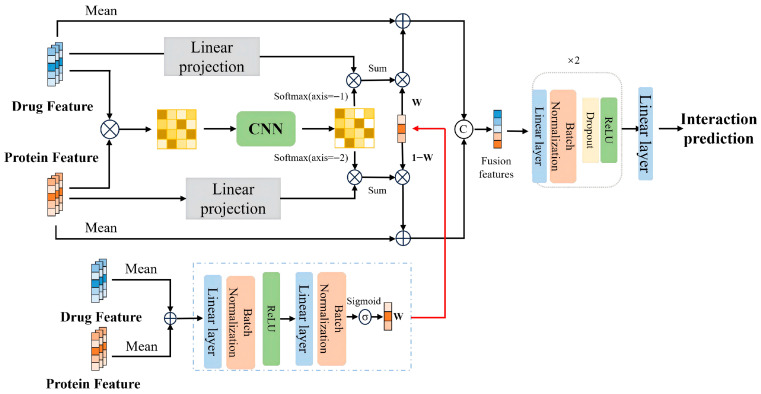
Dual-interaction fusion. Left: a scaled atom–residue similarity field I is computed from ${X_{D}}^{\prime }$ and ${X_{P}}^{\prime }$. Middle: a 2D convolution produces an enhanced field O and directional attentions $A_{D},A_{P}$, yielding context-enriched features $X_{Dm}$ and $X_{Pm}$. Right: channel-wise gating based on $\left(z_{D},z_{P}\right)$ yields w and performs bidirectional residual fusion to obtain global fused representations $z_{D}^{out}$ and $z_{P}^{out}$, which are fed into the MLP prediction head.

To obtain global interaction features, we apply mean pooling to the interaction-informed representations:

(19) $$u_{D}=MeanPool(X_{Dm})=\frac{1}{n}\sum_{i=1}^{n}x_{i}^{\left(Dm\right)},\;\;\;\;\;\;\;\;\;\;\;u_{P}=MeanPool(X_{Pm})=\frac{1}{\ell }\sum_{j=1}^{\ell }x_{j}^{\left(Pm\right)},$$

where $x_{i}^{\left(Dm\right)}$ and $x_{j}^{\left(Pm\right)}$ are the i-th and j-th rows of $X_{Dm}$ and $X_{Pm}$, respectively. We then perform gated residual fusion at the global level:

(20) $$z_{D}^{out}=z_{D}+w⊙u_{D},\;\;\;\;\;\;\;\;\;\;\;z_{P}^{out}=z_{P}+(1-w)⊙u_{P},$$

where ⊙ denotes element-wise multiplication and 1−w is taken element-wise. Large $w_{k}$ values let channel k on the drug side rely more on protein-informed interaction features, while small $w_{k}$ values delegate more interaction information to the protein side via $(1-w{)}_{k}$. This complementary gating yields a stable and interpretable bidirectional fusion mechanism (Figure 4).

#### 3.6.4. Prediction Head

The final fusion representation is formed by concatenating the gated global summaries:

(21) $$x_{f}=[\;z_{D}^{out};z_{P}^{out}\;]\in R^{2m}.$$

A two-layer MLP decoder then maps $x_{f}$ to a scalar logit and interaction probability:

(22) $$s=W_{2}\;\phi (W_{1}x_{f}+b_{1})+b_{2},$$

(23)  p=σ(s)∈[0,1],

where $W_{1}\in R^{h\times 2m}$, $W_{2}\in R^{1\times h}$, $b_{1}\in R^{h}$ and $b_{2}\in R$ are learnable parameters, h is the hidden width of the decoder, ϕ(⋅) is a nonlinearity (e.g., ReLU), and σ(⋅) is the Sigmoid function. All parameters in the encoders, dual-interaction fusion module and decoder are optimized end-to-end with the binary cross-entropy loss.

#### 3.6.5. Summary and Discussion

In summary, the dual-interaction fusion module implements

$$({X_{D}}^{\prime },z_{D},{X_{P}}^{\prime },z_{P})⟼z_{D}^{out},z_{P}^{out},p,$$

where the key innovations are (i) an explicit atom–residue similarity field that can be visualized and further refined by a lightweight 2D neighborhood operator; (ii) bidirectional aggregation that produces drug- and protein-side features enriched with cross-modal context; and (iii) a channel-wise gating mechanism that adaptively balances unimodal and cross-modal information, yielding stable fusion across heterogeneous feature scales.

### 3.7. Rationale for Architectural and Hyperparameter Choices

We chose a moderate depth for the multi-scale GCN encoder to balance receptive-field coverage and the risk of over-smoothing in deeper message passing. Multiple blocks/layers improve neighborhood awareness, while excessive depth may homogenize node representations; thus, we use a width-aligned, moderately deep design.

For the protein encoder, multi-head self-attention is configured to keep the per-head dimension in a stable range and to encourage diverse subspace projections, which improves optimization stability and representation richness.

For the **2D neighborhood refinement** over the atom–residue similarity field, we adopt a **small kernel (e.g., 3 × 3)** to capture local continuity along both atom and residue neighborhoods while avoiding oversmoothing sparse interaction patterns and unnecessary computation. Overall, hyperparameters were finalized on the validation set during development and kept fixed for all comparisons.

The concrete hyperparameter settings are summarized in Table 2.

## 4. Conclusions

This paper presents GADFDTI, a novel deep learning framework for drug–target interaction (DTI) prediction that integrates multi-scale graph neural networks, attention- based protein encoders, and an attention-guided dual-interaction fusion mechanism. By jointly capturing local and global features from both drugs and proteins, and explicitly modeling fine-grained atomic-residue alignments, our model effectively addresses key challenges in cross-modal representation learning and interaction modeling. Extensive experiments on two benchmark datasets (**Human** and ***C. elegans***) demonstrated that GADFDTI outperforms most of the state-of-the-art DTI prediction models across multiple evaluation metrics. Ablation studies further confirmed the effectiveness of each architectural component, particularly the synergy between attention and convolution in the fusion module. Furthermore, a case study on SARS-CoV-2 antiviral drug repositioning showed that GADFDTI assigns high confidence scores to compounds such as remdesivir and ritonavir, which have demonstrated clinical efficacy [25,26], while correctly rejecting Aspirin as a non-interacting agent. This suggests that our model has practical potential to serve as a computational prescreening tool in drug discovery pipelines, especially in urgent scenarios such as emerging infectious diseases.

## Figures and Tables

**Table 1:** Dataset statistics.

Dataset	Drugs	Targets	Pairs	Positive	Negative
Human	2726	2001	6728	3364	3364
*C. elegans*	1767	1876	7786	3893	3893

**Table 2:** Training and model hyperparameters.

Parameter	Setting
Atom feature dimension	87
Max protein length	1200
Protein embedding dim.	128
Multi-GCN blocks	3
GCN layers per block	4
linear transition layers	3
MSSAN mask windows	[0, 3, 12, 1200]
Protein encoder layers	2
Fusion CNN layers	3
Fusion kernel size	3
Batch size	128
Epochs	100
Optimizer	Adam
Learning rate	0.0001
GPU memory usage	2 × 24 GB

**Table 3:** Effect of adding a protein-side 1D-CNN on **Human** and ***C. elegans***. (The bolded value represents the best result among the unified indicators. The following tables are the same).

Dataset	Method	AUC	Precision	Recall
Human	Baseline (MGNN + MSSAN)	0.984	0.951	0.947
	+1D-CNN	**0.985**	**0.955**	**0.953**
*C. elegans*	Baseline (MGNN + MSSAN)	**0.995**	0.964	0.959
	+1D-CNN	0.994	**0.969**	**0.961**

**Table 4:** Ablation of gating and dot-product within the dual interaction.

Dataset	Method	AUC	Precision	Recall
Human	Baseline + 1D-CNN	0.985	0.955	0.953
	+Gating only	0.984	0.953	0.956
	+Dot-product only	0.980	0.945	0.951
	GADFDTI	**0.986**	**0.966**	**0.960**
*C. elegans*	Baseline + 1D-CNN	0.994	0.969	0.961
	+Gating only	0.993	0.969	0.956
	+Dot-product only	0.986	0.955	0.961
	GADFDTI	**0.996**	**0.975**	**0.967**

**Table 5:** Effect of removing the neighborhood filter from the dual interaction.

Dataset	Method	AUC	Precision	Recall
Human	GADFDTI	**0.986**	**0.966**	**0.960**
	-Neighborhood	0.983	0.959	0.956
*C. elegans*	GADFDTI	**0.996**	**0.975**	**0.967**
	-Neighborhood	0.994	0.971	0.962

**Table 6:** Fusion variants versus GADFDTI on **Human** and ***C. elegans***.

Fusion	Human			*C. elegans*		
	AUC	Prec.	Rec.	AUC	Prec.	Rec.
Baseline + 1D-CNN (concat)	0.985	0.955	0.953	0.994	0.969	0.956
Cross attention	0.967	0.912	0.950	0.981	0.961	0.942
Bi-intent attention	0.977	0.954	0.947	0.991	0.977	0.945
Union attention	0.973	0.921	0.941	0.991	0.972	0.950
Bilinear attention	0.978	0.954	0.938	0.990	0.931	0.962
GADFDTI	**0.986**	**0.966**	**0.960**	**0.996**	**0.975**	**0.967**

**Table 7:** Classification comparison of GADFDTI against recent baselines on two benchmarks.

Method	Human			*C. elegans*		
	AUC	Precision	Recall	AUC	Precision	Recall
TransformerCPI	0.972	0.938	0.932	0.984	0.943	0.951
MHSADTI	0.982	0.947	0.937	0.984	0.947	0.945
CoaDTI	0.974	0.922	0.950	0.983	0.944	0.943
Mutual-DTI	0.984	0.962	0.943	0.987	0.948	0.949
MdDTI	**0.989**	0.956	0.947	0.994	0.964	**0.970**
MultiGranDTI	0.978	0.926	0.956	0.986	0.963	0.951
**GADFDTI**	0.986	**0.966**	**0.960**	**0.996**	**0.975**	0.967

**Table 8:** Predicted interaction probabilities for SARS-CoV-2 targets.

Pair	Probability	Expected Label
3CL_pro_–Baricitinib	0.994	positive
3CL_pro_–Remdesivir	0.972	positive
3CL_pro_–Ritonavir	0.972	positive
3CL_pro_–Lopinavir	0.850	positive
3CL_pro_–Aspirin	0.030	negative
RdRp–Ivermectin	0.993	positive
RdRp–Sofosbuvir	0.979	positive
RdRp–Remdesivir	0.950	positive
RdRp–Daclatasvir	0.847	positive
RdRp–Lopinavir	0.812	positive
RdRp–Ritonavir	0.538	positive
RdRp–Aspirin	0.070	negative

## Data Availability

All data analyzed in this study are derived from previously published, publicly available drug–target interaction resources. The Human and *C. elegans* benchmark datasets used in our experiments are constructed from known drug–target interaction pairs curated in the Matador/SuperTarget and DrugBank databases. The underlying databases can be accessed at: Matador (Manually Annotated Targets and Drugs Online Resource): https://pmc.ncbi.nlm.nih.gov/articles/PMC4765858/ (accessed on 27 October 2025). SuperTarget: http://insilico.charite.de/supertarget (accessed on 7 August 2025). DrugBank: https://go.drugbank.com (accessed on 7 August 2025). Credible negative samples are generated following the strategy proposed by Liu et al. [13]. The exact train/validation/test splits and preprocessed feature files used in this work can be obtained from the corresponding author upon reasonable request.

## Supplementary Materials

The following supporting information can be downloaded at: https://www.mdpi.com/article/10.3390/molecules31030498/s1, Supplementary Table S1: Paired *t*-test results across 10 matched random seeds (two-sided, *n* = 10, α = 0.05) comparing GADFDTI with baseline methods. AUC is reported as mean ± standard deviation across seeds. Mean difference = (GADFDTI—baseline). *p*-values reported as “*p* < 1 × 10^−4^” when printed as 0.0000 in logs; Supplementary Table S2. Sliding-window aggregation for long proteins (inference only). Proteins longer than 1200 residues are evaluated on the >1200 subset using two inference strategies: (i) truncation to the first 1200 residues; (ii) sliding-window inference with window length = 1200, stride = 300, and max pooling over window-level scores. Values are reported as mean ± standard deviation over 10 random seeds.

## Abbreviations

The following abbreviation is used in this manuscript:

GADFDTI	Gated-Attention Dual-Fusion Drug-Target Interaction
