# Supplementary material for: Drug–Target Interaction Prediction via Dual-Interaction Fusion
Source: Molecules. 2026 Jan 31;31(3):498. doi: 10.3390/molecules31030498 (PMC12899975; doi:10.3390/molecules31030498)
Supplement: Supplementary file 1 [file molecules-31-00498-s001.zip › molecules-4067852-supplementary.pdf]

Supplementary Materials

Supplementary Table S1: Paired *t*-test results across 10 matched random seeds (two-sided, n = 10, α = 0.05) comparing GADFDTI with baseline methods. AUC is reported as mean ± standard deviation across seeds. Mean difference = (GADFDTI—baseline). *p*-values reported as "$p < 1 \times 10^{-4}$" when printed as 0.0000 in logs;

Table S1(a) Human benchmark

Compared method	AUC (mean±std)	GADFDTI AUC (mean±std)	Mean diff	t	p-value	Sig. (α=0.05)
Baseline+1D-CNN	0.9841 ± 0.0017	0.9866 ± 0.0020	0.0025	3.000	0.0150	Yes
+Gating only	0.9848 ± 0.0011	0.9866 ± 0.0020	0.0018	2.862	0.0187	Yes
+Dot-product only	0.9794 ± 0.0014	0.9866 ± 0.0020	0.0072	9.900	< 1e-4	Yes
-Neighborhood	0.9839 ± 0.0022	0.9866 ± 0.0020	0.0027	2.793	0.0209	Yes
Cross attention	0.9664 ± 0.0019	0.9866 ± 0.0020	0.0202	27.776	< 1e-4	Yes
Bi-intent attention	0.9762 ± 0.0019	0.9866 ± 0.0020	0.0104	12.911	< 1e-4	Yes
Union attention	0.9723 ± 0.0021	0.9866 ± 0.0020	0.0143	14.453	< 1e-4	Yes
Bilinear attention	0.9770 ± 0.0019	0.9866 ± 0.0020	0.0096	12.829	< 1e-4	Yes
TransformerCPI	0.9713 ± 0.0016	0.9866 ± 0.0020	0.0153	19.043	< 1e-4	Yes
MHSADTI	0.9828 ± 0.0020	0.9866 ± 0.0020	0.0038	3.726	0.0047	Yes
CoaDTI	0.9738 ± 0.0025	0.9866 ± 0.0020	0.0128	10.064	< 1e-4	Yes
Mutual-DTI	0.9844 ± 0.0021	0.9866 ± 0.0020	0.0022	2.071	0.0683	No
MultiGranDTI	0.9777 ± 0.0022	0.9866 ± 0.0020	0.0089	9.273	< 1e-4	Yes
MdDTI	0.9879 ± 0.0016	0.9866 ± 0.0020	-0.0013	-1.494	0.1693	No

Table S1(b) C. elegans benchmark

Compared method	AUC (mean±std)	GADFDTI AUC (mean±std)	Mean diff	t	p-value	Sig. (α=0.05)
Baseline+1D-CNN	0.9948 ± 0.0023	0.9962 ± 0.0020	0.0014	1.288	0.2300	No
+Gating only	0.9932 ± 0.0022	0.9962 ± 0.0020	0.0030	2.620	0.0278	Yes
+Dot-product only	0.9859 ± 0.0019	0.9962 ± 0.0020	0.0103	13.538	< 1e-4	Yes
-Neighborhood	0.9950 ± 0.0011	0.9962 ± 0.0020	0.0012	1.527	0.1612	No
Cross attention	0.9806 ± 0.0022	0.9962 ± 0.0020	0.0156	21.273	< 1e-4	Yes
Bi-intent attention	0.9899 ± 0.0015	0.9962 ± 0.0020	0.0063	10.549	< 1e-4	Yes
Union attention	0.9915 ± 0.0018	0.9962 ± 0.0020	0.0047	4.413	0.0017	Yes
Bilinear attention	0.9905 ± 0.0020	0.9962 ± 0.0020	0.0057	8.143	< 1e-4	Yes
TransformerCPI	0.9845 ± 0.0016	0.9962 ± 0.0020	0.0117	14.562	< 1e-4	Yes
MHSADTI	0.9853 ± 0.0020	0.9962 ± 0.0020	0.0109	11.792	< 1e-4	Yes
CoaDTI	0.9823 ± 0.0017	0.9962 ± 0.0020	0.0139	13.260	< 1e-4	Yes
Mutual-DTI	0.9873 ± 0.0016	0.9962 ± 0.0020	0.0089	10.027	< 1e-4	Yes
MultiGranDTI	0.9855 ± 0.0022	0.9962 ± 0.0020	0.0107	10.815	< 1e-4	Yes
MdDTI	0.9969 ± 0.0017	0.9962 ± 0.0020	-0.0007	-1.413	0.1914	No

Supplementary Table S2. Sliding-window aggregation for long proteins (inference only). Proteins longer than 1200 residues are evaluated on the >1200 subset using two inference strategies: (i) truncation to the first 1200 residues; (ii) sliding-window inference with window length = 1200, stride = 300, and max pooling over window-level scores. Values are reported as mean ± standard deviation over 10 random seeds.

Dataset	Subset	Inference strategy	AUC	AUPR	F1
Human	>1200	Truncation (first 1200)	0.9840 ± 0.0030	0.9831 ± 0.0033	0.951 ± 0.009
Human	>1200	Sliding-window + max	0.9853 ± 0.0028	0.9847 ± 0.0030	0.954 ± 0.010
C. elegans	>1200	Truncation (first 1200)	0.9949 ± 0.0027	0.9942 ± 0.0029	0.961 ± 0.010
C. elegans	>1200	Sliding-window + max	0.9952 ± 0.0025	0.9947 ± 0.0027	0.967 ± 0.009
